# Supplementary material for: Divergent regulation of KCNQ1/E1 by targeted recruitment of protein kinase A to distinct sites on the channel complex
Source: eLife. 2023 Aug 31;12:e83466. doi: 10.7554/eLife.83466 (PMC10499372; doi:10.7554/eLife.83466)

Figure 2B

Anti-phosphoQ1 immunoblot

1: Q1-YFP + E1 + nano

2: Q1-YFP + E1 + nanoCα

3: Q1 + E1-YFP + pcDNA3

4: Q1 + E1-YFP + free Cα

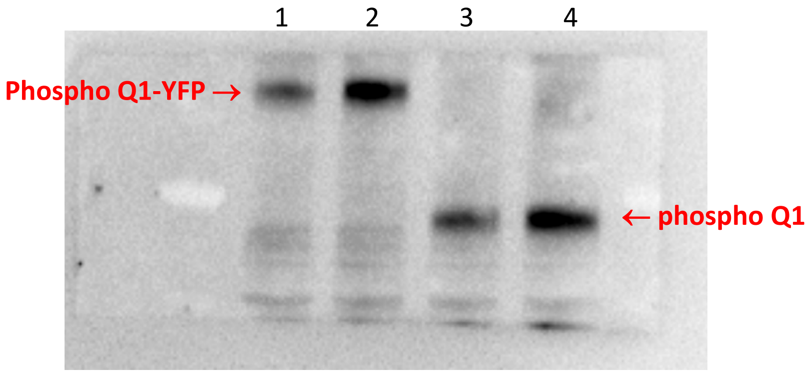

Figure 2B

Anti-actin immunoblot

1: Q1-YFP + E1 + nano

2: Q1-YFP + E1 + nanoCα

3: Q1 + E1-YFP + pcDNA3

4: Q1 + E1-YFP + free Cα

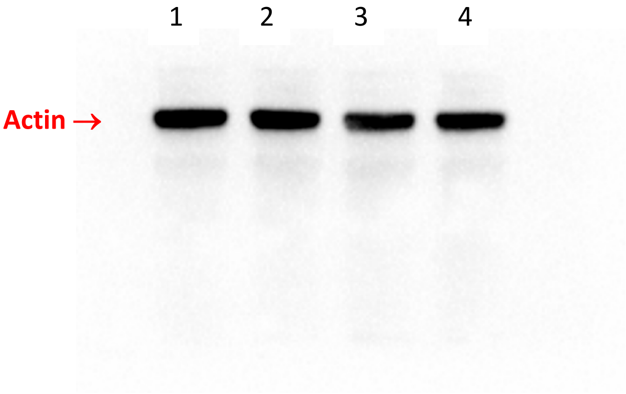

Figure 2B

Anti-Q1 immunoblot

1: Q1-YFP + E1 + nano

2: Q1-YFP + E1 + nanoCα

3: Q1 + E1-YFP + pcDNA3

4: Q1 + E1-YFP + free Cα

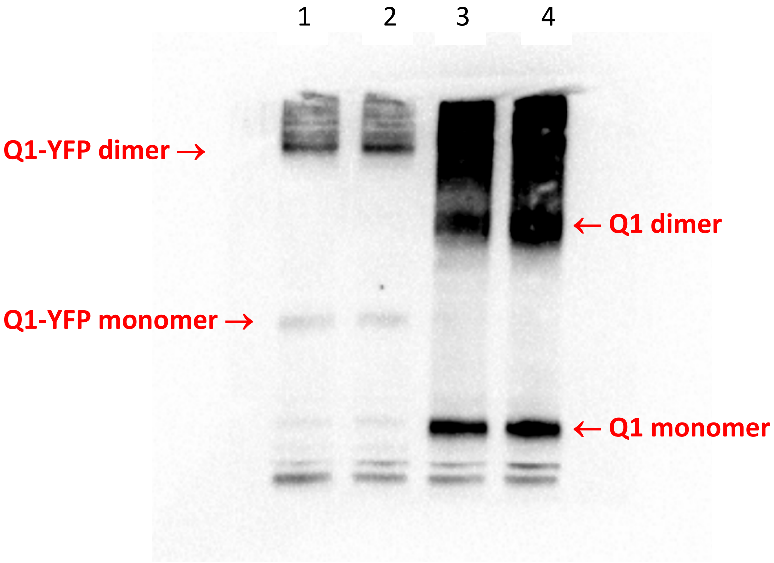

Figure 2F

Anti-phosphoQ1 immunoblot

1: Q1 + E1-YFP + nanoCα

2: Q1 + E1-YFP + nano

3: Q1 + E1-YFP + nano + yotiao

4: Q1 + E1-YFP + free Cα

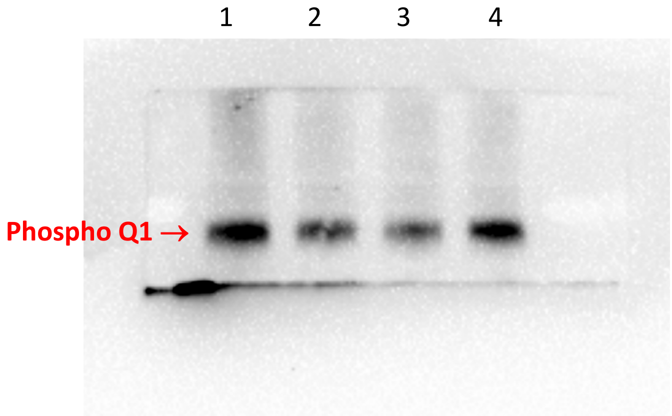

Figure 2F

Anti-actin immunoblot

1: Q1 + E1-YFP + nanoCα

2: Q1 + E1-YFP + nano

3: Q1 + E1-YFP + nano + yotiao

4: Q1 + E1-YFP + free Cα

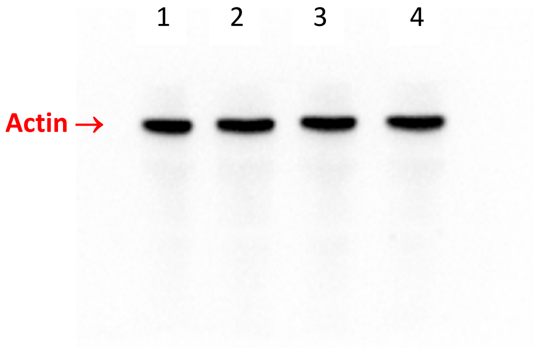

Figure 2F

Anti-Q1 immunoblot

1: Q1 + E1-YFP + nanoCα

2: Q1 + E1-YFP + nano

3: Q1 + E1-YFP + nano + yotiao

4: Q1 + E1-YFP + free Cα

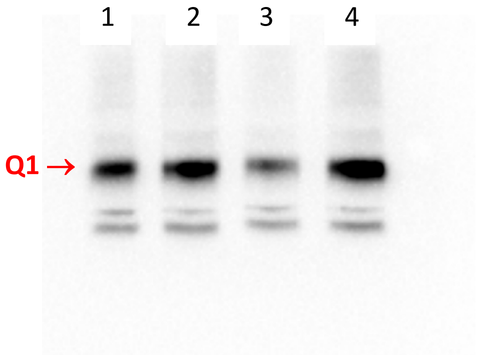

Supplement: Figure 2—source data 2. [file elife-83466-fig2-data2.zip › Figure 2 - source data 2/Figure 2 - source data 2.pdf]
