# Supplementary figures and images for: Divergent regulation of KCNQ1/E1 by targeted recruitment of protein kinase A to distinct sites on the channel complex
Source: eLife. 2023 Aug 31;12:e83466. doi: 10.7554/eLife.83466 (PMC10499372; doi:10.7554/eLife.83466)

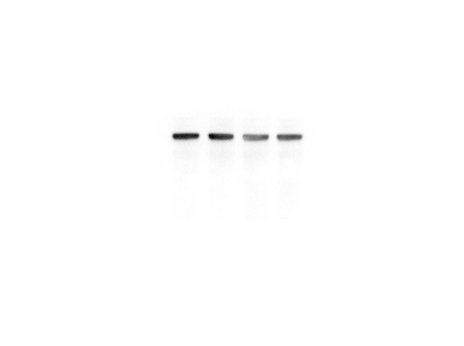

Supplement: Figure 2—source data 2. [file elife-83466-fig2-data2.zip › Figure 2B - Actin.jpg]

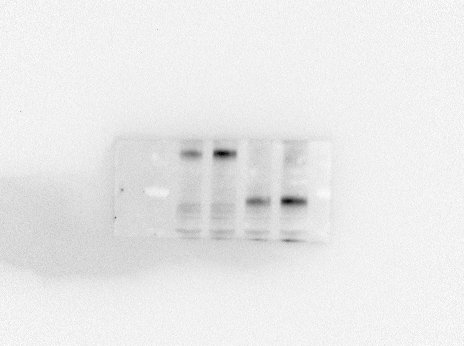

Supplement: Figure 2—source data 2. [file elife-83466-fig2-data2.zip › Figure 2B - phosphoQ1.jpg]

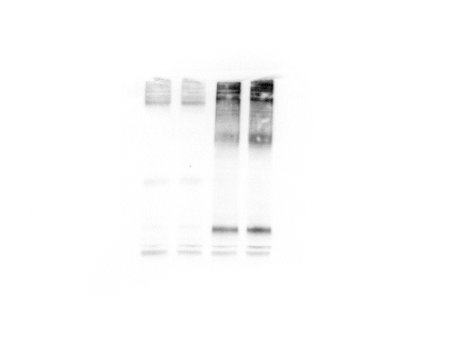

Supplement: Figure 2—source data 2. [file elife-83466-fig2-data2.zip › Figure 2B - Q1.jpg]

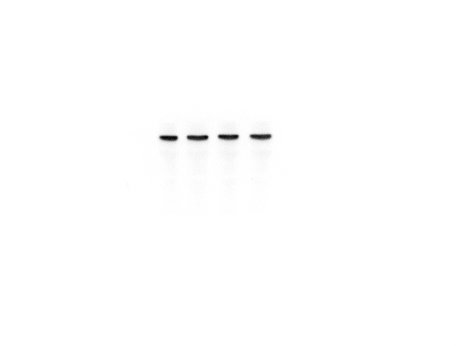

Supplement: Figure 2—source data 2. [file elife-83466-fig2-data2.zip › Figure 2F - Actin.jpg]

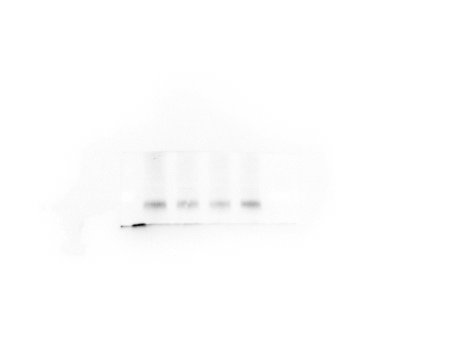

Supplement: Figure 2—source data 2. [file elife-83466-fig2-data2.zip › Figure 2F - phosphoQ1.jpg]

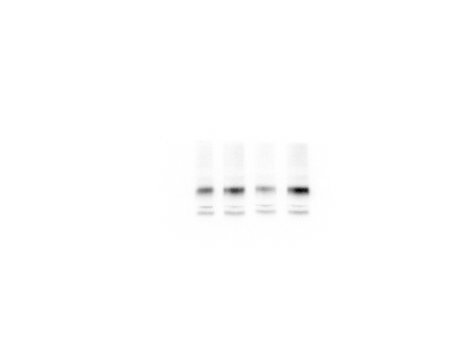

Supplement: Figure 2—source data 2. [file elife-83466-fig2-data2.zip › Figure 2F - Q1.jpg]

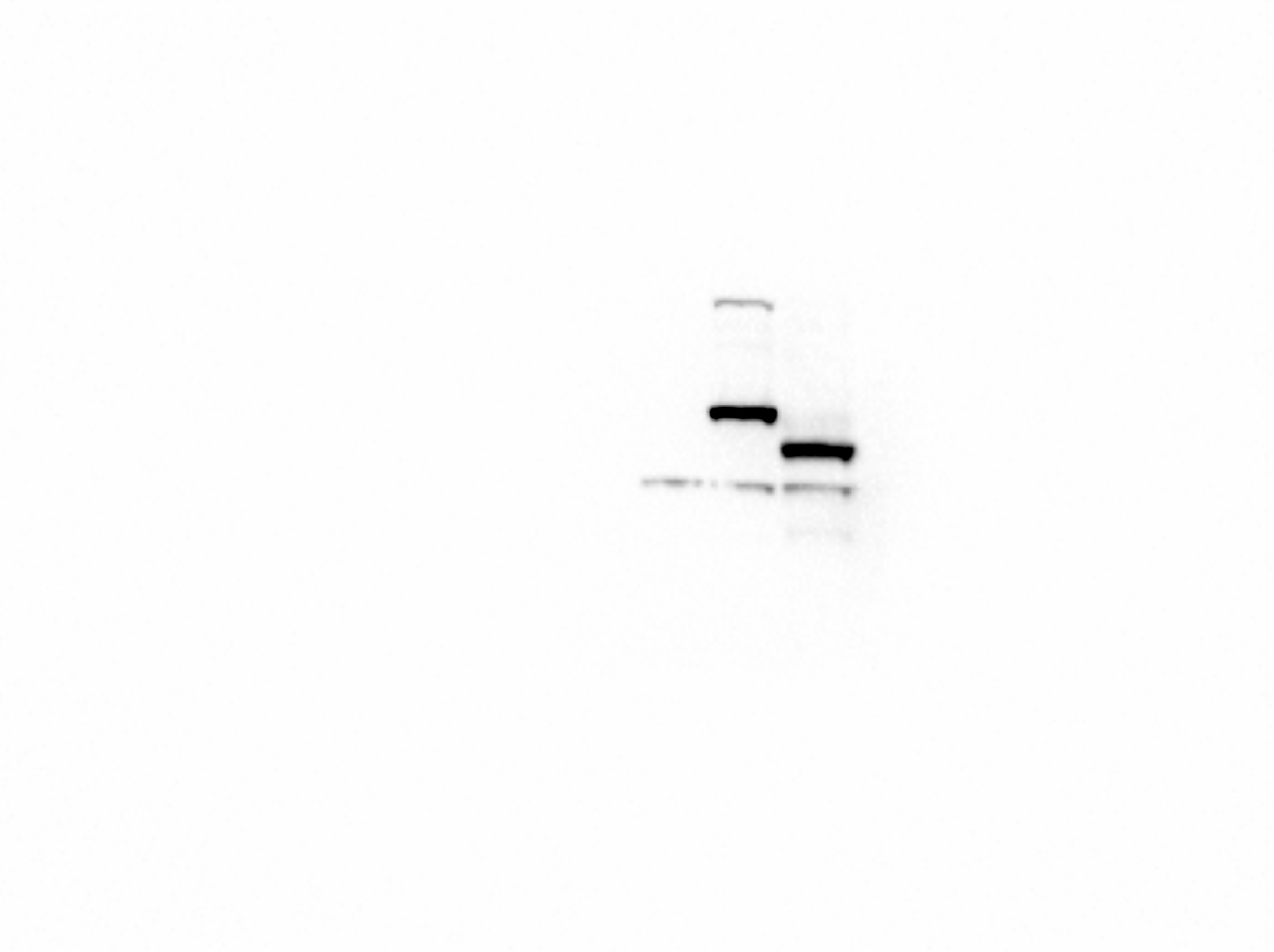

Supplement: Figure 2—figure supplement 1—source data 1. [file elife-83466-fig2-figsupp1-data1.zip › IP_ut Q1E1YFPnanoca Q1E1yfppka_antica2.tif]

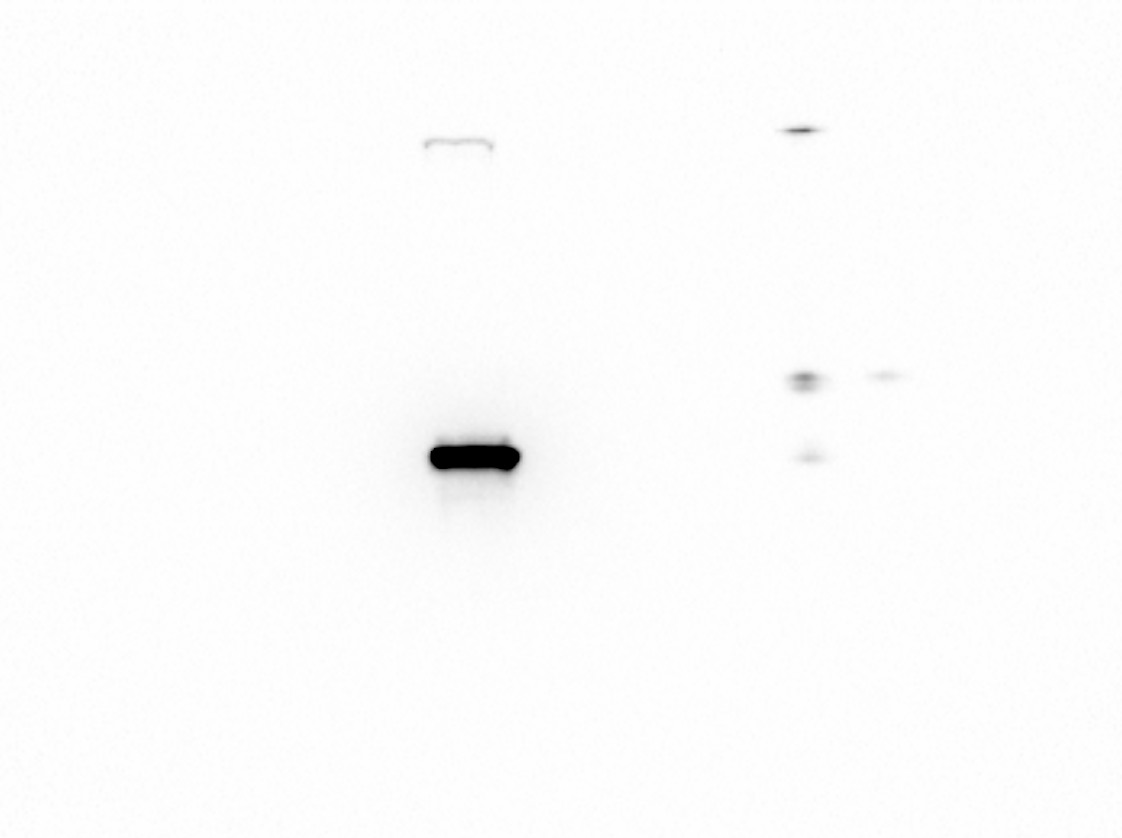

Supplement: Figure 2—figure supplement 1—source data 1. [file elife-83466-fig2-figsupp1-data1.zip › IP_ut Q1E1YFPnanoca Q1E1yfppka_antiYFP_WB_UT q1e1yfpnanoca Q1E1yfppka_antiyfp1.tif]

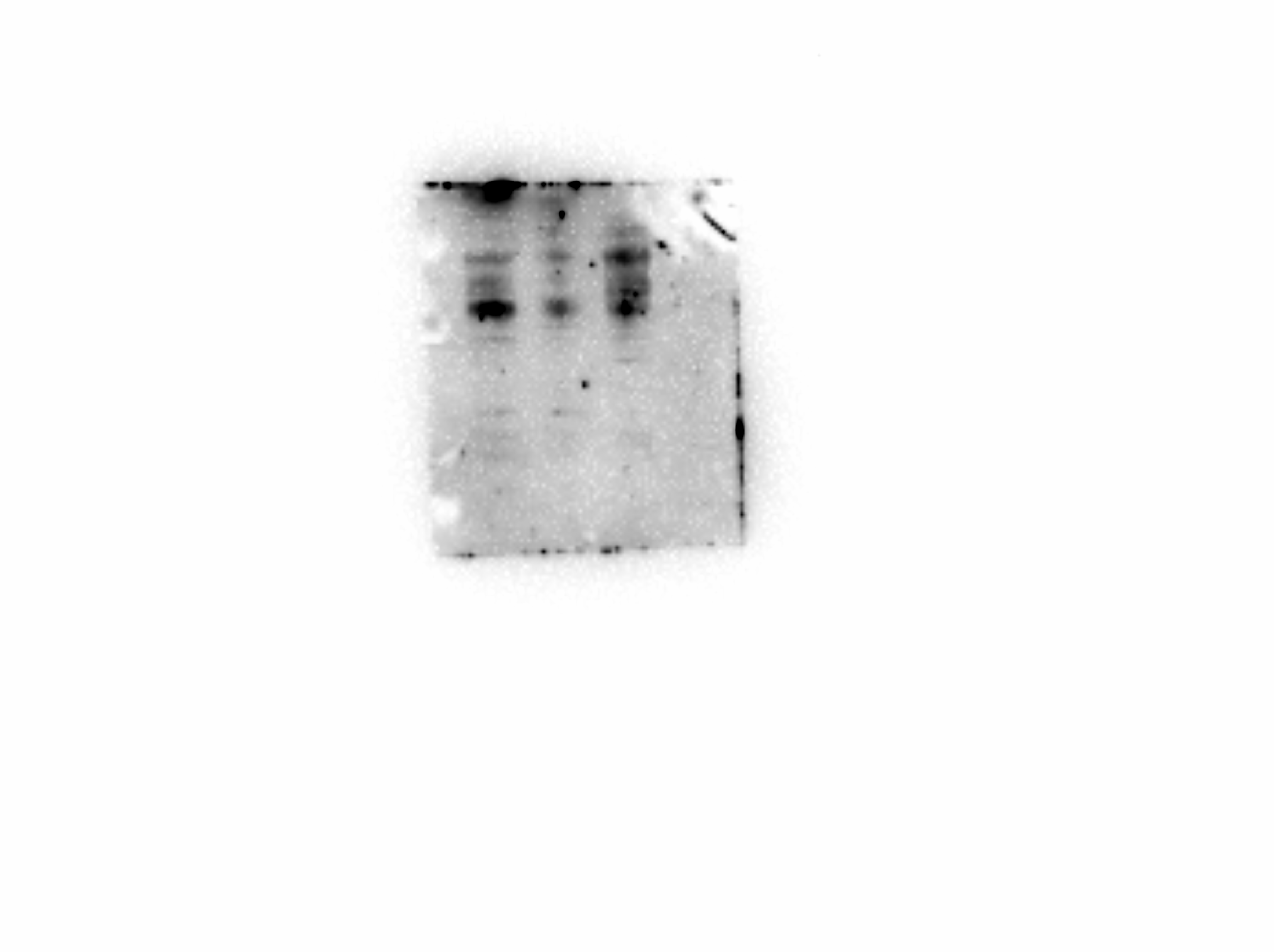

Supplement: Figure 2—figure supplement 1—source data 1. [file elife-83466-fig2-figsupp1-data1.zip › WB_ut Q1E1YFPnanoca Q1E1yfppka_antiactin.tif]
