# Supplementary material for: Divergent regulation of KCNQ1/E1 by targeted recruitment of protein kinase A to distinct sites on the channel complex
Source: eLife. 2023 Aug 31;12:e83466. doi: 10.7554/eLife.83466 (PMC10499372; doi:10.7554/eLife.83466)
Supplement: Figure 2—figure supplement 1—source data 1. [file elife-83466-fig2-figsupp1-data1.zip › Figure 2 - figure supplement 1 full gels.docx]

**Figure 2 – figure supplement 1**

**Anti-YFP immunoblot (PKA pull-down) Anti-PKA immunoblot (PKA pull-down)**

**Lanes:**

1. **Untransfected control**
2. **Q1 E1-YFP nanoCa**
3. **Q1 E1-YFP PKA-Ca**

11

21

31

31

21

11

**
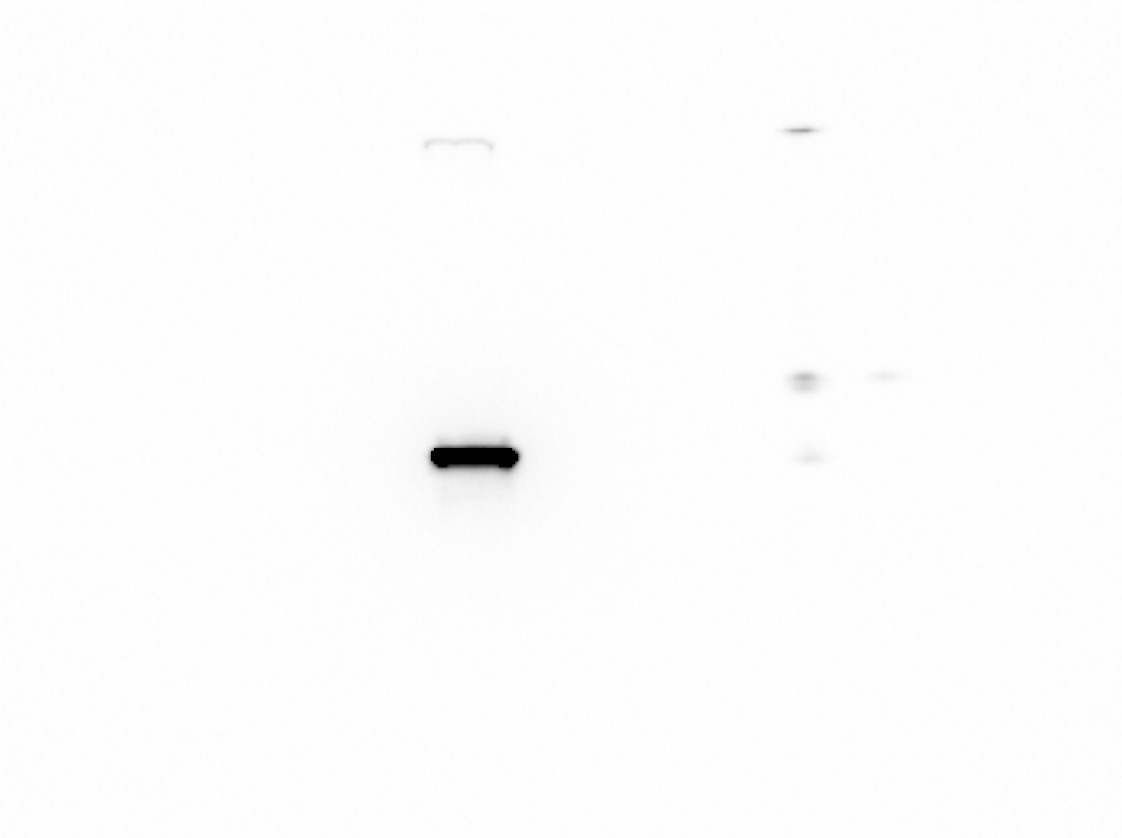
**

**nanoCa**

**PKA-Ca**

**E1-YFP →**

**Anti-YFP immunoblot (input western) Anti-actin immunoblot (input western)**

**Lanes:**

1. **Untransfected control**
2. **Q1 E1-YFP nanoCa**
3. **Q1 E1-YFP PKA-Ca**

**
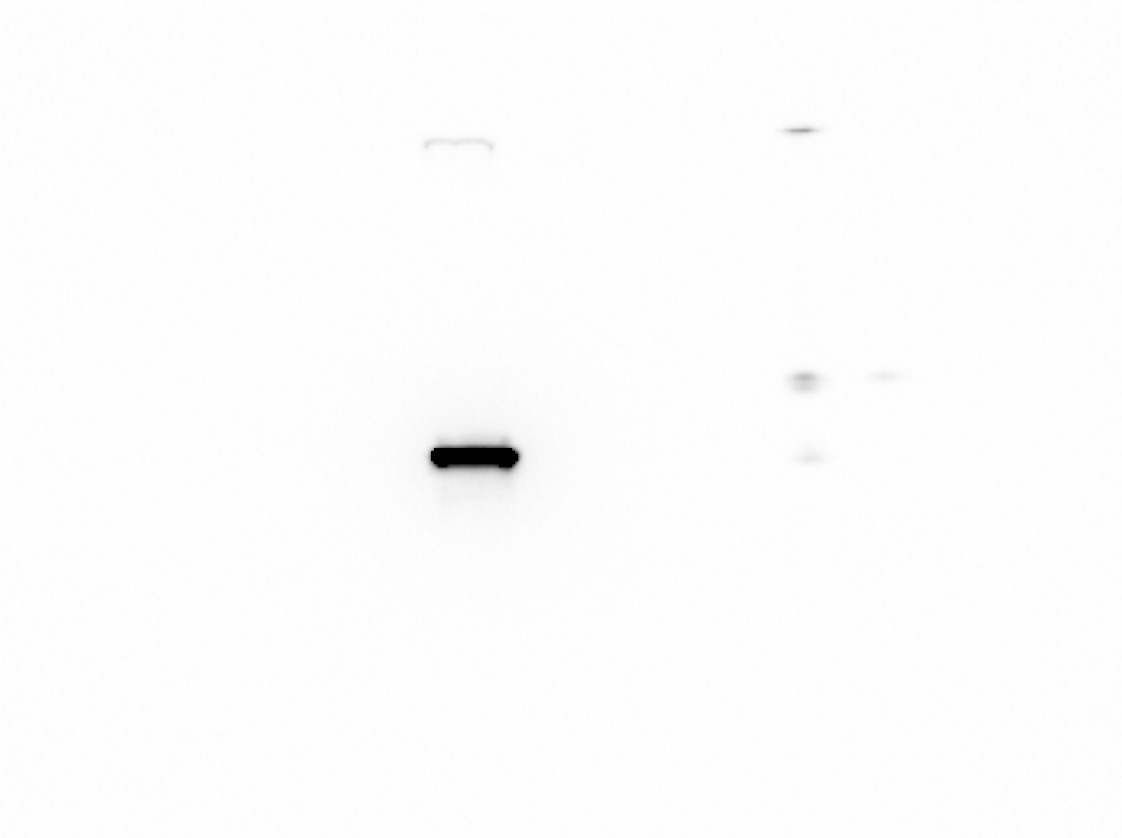
**

11

21

31

1

11

21

31

1

**E1-YFP →**

**Actin →**
